# A novel paper MAP method for rapid high resolution histological analysis

**DOI:** 10.1038/s41598-021-02632-1

**Published:** 2021-12-02

**Authors:** Mirae Lee, Jiwon Woo, Doh-Hee Kim, Yu-Mi Yang, Eunice Yoojin Lee, Jung-Hee Kim, Seok-Gu Kang, Jin-Kyung Shim, Jeong-Yoon Park

**Affiliations:** 1 Department of Neurosurgery, Graduate School of Medical Science, Brain Korea 21 Project, Yonsei University College of Medicine, Seoul, 03722 Republic of Korea; 2 The Spine and Spinal Cord Institute, Department of Neurosurgery, Gangnam Severance Hospital, Yonsei University College of Medicine, Seoul, 06273 Republic of Korea; 3 Biomedical Research Center, Gangnam Severance Hospital, Yonsei University College of Medicine, Seoul, 06273 Republic of Korea; 4 Biomedical Research Institute, Biohedron Therapeutics Co., Ltd, Seoul, 06273 Republic of Korea; 5 Research Institute, Seoul Medical Center, Seoul, 02053 Republic of Korea; 6 Department of Neurosurgery, Brain Tumor Center, Severance Hospital, Yonsei University College of Medicine, Seoul, 03722 Republic of Korea; 7 Department of Medical Sciences, Yonsei University Graduate School, Seoul, 03722 Republic of Korea; 8 Columbia University Vagelos College of Physicians and Surgeons, New York, NY 10032 USA

**Keywords:** Biological techniques, Biotechnology, Neuroscience, Diseases, Molecular medicine, Neurology

## Abstract

Three-dimensional visualization of cellular and subcellular-structures in histological-tissues is essential for understanding the complexities of biological-phenomena, especially with regards structural and spatial relationships and pathologlical-diagnosis. Recent advancements in tissue-clearing technology, such as Magnified Analysis of Proteome (MAP), have significantly improved our ability to study biological-structures in three-dimensional space; however, their wide applicability to a variety of tissues is limited by long incubation-times and a need for advanced imaging-systems that are not readily available in most-laboratories. Here, we present optimized MAP-based method for paper-thin samples, Paper-MAP, which allow for rapid clearing and subsequent imaging of three-dimensional sections derived from various tissues using conventional confocal-microscopy. Paper-MAP successfully clear tissues within 1-day, compared to the original-MAP, without significant differences in achieved optical-transparency. As a proof-of-concept, we investigated the vasculature and neuronal-networks of a variety of human and rodent tissues processed via Paper-MAP, in both healthy and diseased contexts, including Alzheimer’s disease and glioma.

## Introduction

The development of CLARITY (Clear Lipid-exchanged Acrylamide-hybridized Rigid Imaging/Immunostaining/In situ hybridization-compatible Tissue hYdrogel) and subsequent tissue clearing protocols have allowed for the three-dimensional visualization of biological structures in intact tissues at an unprecedented resolution^1–7^. Compared to conventional methods for histopathological assessment of biological tissues, CLARITY-based techniques show improved preservation of tissue structure, protein localization, and molecular states^3,4,8^. Many of these techniques involve physically expanding biological samples embedded in swellable gels to overcome the resolution limit of conventional light microscopy. A recently developed tissue clearing and expansion technique, Magnified Analysis of Proteome (MAP), has demonstrated improved preservation of three-dimensional proteome and structural organization, particularly in thick tissues^9^.

Nonetheless, these approaches often require advanced imaging methods such as expansion microscopy (ExM)^10^, ultrastructure expansion microscopy (U-ExM)^11^ and SmartSPIM^12^. Standard confocal microscopes have a limited working distance and cannot accommodate high degrees of tissue expansion, these tissue clearing and imaging approaches cannot be easily performed in standard laboratories and human histological tissue, limiting their widespread use.

Here, we introduce novel MAP-based clearing techniques, “Paper-MAP”, which enable multiscale super-resolution imaging of biological structures while overcoming the aforementioned limitations of previously published approaches. Paper-MAP technique involve using paper-thin sections of harvested tissue for clearing, expansion, and subsequent imaging. Specifically, Paper-MAP involves generating a tissue-hydrogel hybrid that is less than 100-µm in thickness for stable super-resolution imaging, which requires only 2 days from tissue harvesting to immunostaining. As a proof-of-concept, we demonstrate the use of Paper-MAP to investigate vasculature and neuronal networks within rodent and human brain tissue, in both healthy and diseased states.

## Results

### Paper-MAP: A method for rapid tissue clearing and expansion for three-dimensional ultrastructural imaging of intact tissues

**Figure 1:**
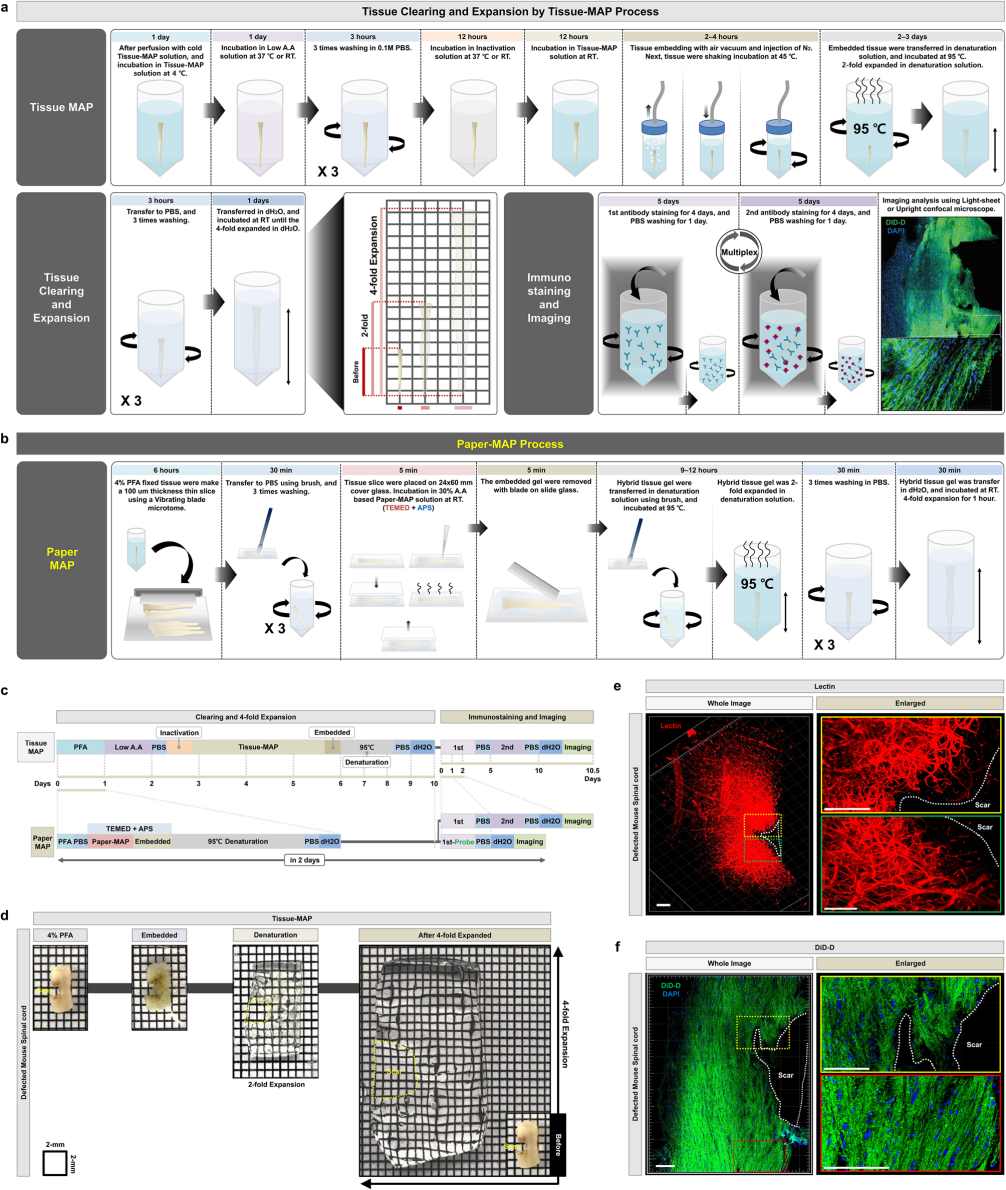
Tissue clearing and expansion via Tissue-MAP. (**a**) Schematic representation of Tissue-MAP protocol for clearing, expanding, and subsequent immunostaining of whole tissue. (**b**) Schematic representation of Paper-MAP processing using 100 μm-thick tissue sections. (**c**) Timeline of Tissue-MAP and Paper-MAP. (**d**) Optical transparency of injured mouse spinal cord achieved via Tissue-MAP. Tissue transparency was assessed against a patterned background (length:width = 5 mm:5 mm). (**e** and** f**) Three-dimensional rendering of lectin (red) and DiD-D (green) immunostaining of injured mouse spinal cord tissue processed via Tissue-MAP, imaged with light-sheet microscopy (range: 2000-μm) and confocal microscopy (tile-scanned single-z image). Dotted white lines indicate scar region. Scale bar, 100-μm. Illustration of mechanism is visualized by PowerPoint v2016.

The original MAP protocol (Fig. 1a) successfully allows for tissue clearing and subsequent visualization, as demonstrated by the visualization of thinner, disconnected blood vessel patterns (lectin dye) and neuronal networks (DiD-D dye) within injured mouse spinal cord tissue (Fig. 1d–f, Supplementary Video S1 online). However, its wide applicability is limited by the long time required for tissue processing (e.g., clearing and expansion alone requires a minimum of 10 days, followed by an additional 10 days for immunostaining, as shown in Fig. 1a,c), as well as the working distance of commonly used microscopes, the majority of which measure ~ 2 mm and therefore are not amenable to the imaging of many intact whole tissue samples. Furthermore, a key step in MAP involves whole perfusion of the euthanized mouse with MAP prior to harvesting the tissue of interest, which is not amenable to processing patient biopsies. Additionally, the denaturation step, which requires sample incubation at a high temperature, easily damages tissues prior to imaging.

To address these limitations, we developed Paper-MAP, which enables the rapid clearing and expansion of 100 µm-thick samples in 1 day, and subsequent immunostaining and imaging analysis within a total of 2–3 days (Fig. 1b,c). Key modifications include (1) direct use of samples fixed in 4% PFA, without whole-animal perfusion with MAP solution, and (2) rapid embedding, hybridization, and dissociation with a Paper-MAP cocktail solution consisting of Ammonium persulfate (APS) and Tetramethylethylenediamine (TEMED). Paper-MAP also uses an APS and TEMED-based solution for embedding as opposed to the V50 cationic azo initiator used in the original MAP protocol. In the presence of TEMED in Paper-MAP cocktail solution, TEMED is responsible for the formation of free radicals from persulfate, which allows rapid acrylamide polymerization process of polyacrylamide gels^7,13^. As such, Paper-MAP does not require tissue incubation in inactivation solution or embedding in nitrogen gas, and ultimately leads to a significant reduction in the time required for denaturation and expansion, as well as the time required for subsequent immunostaining.

**Figure 2:**
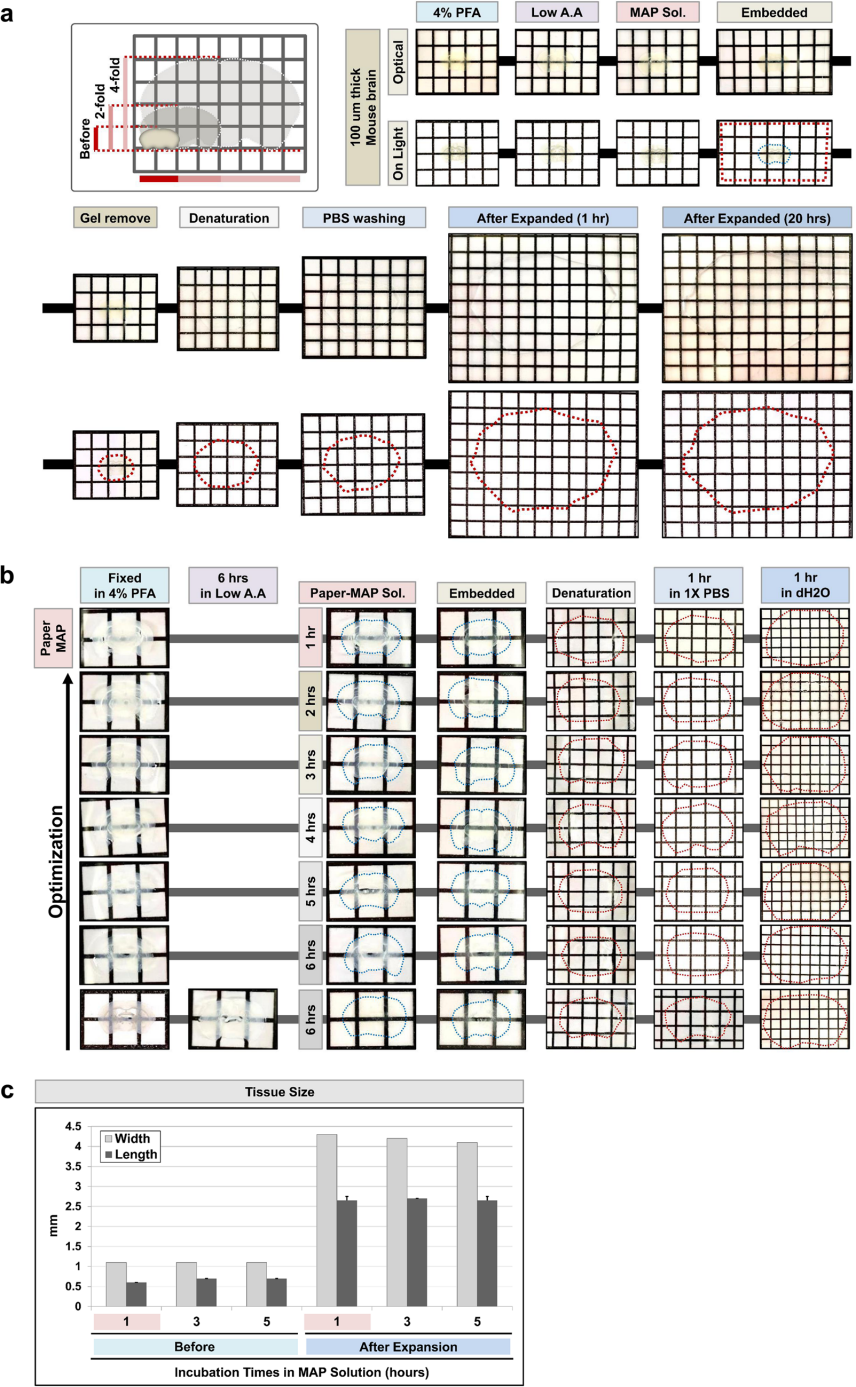
Comparison of tissue clearing and expansion via optimized Paper-MAP. (**a**) Optical transparency of 100 μm-thick mouse brain sections processed via Paper-MAP optimization. (**b**) Effect of varying incubation times in low acrylamide (A.A) solution and Paper-MAP solution on tissue transparency, using 100 μm-thick rodent brain sections. (**c**) Comparison of gel size (width and length) before and after expansion, with varying incubation times in Paper-MAP solution. Quantification of total width and length from different ratio combinations. Data are presented as mean ± SD (standard deviation); n = 5 for each experimental group.

To demonstrate the feasibility and efficacy of optimized Tissue-MAP, we processed mouse brain slices according to the optimization of Tissue-MAP protocol outlined in Fig. 2a. To further optimize and reduce the incubation times required for tissue processing, we optimized Tissue-MAP protocol, which removes the Low acrylamide (A.A) solution incubation step and requires only one hour for hybridization in Paper-MAP solution, as opposed to 3 days in Tissue-MAP (Fig. 2b). As shown in Fig. 2c, these modifications did not result in significant differences in achieved tissue transparency, but nonetheless achieved clearing and expansion in less than 24 h. In addition to a reduction in sample processing time, Paper-MAP allow for the preservation of hard-to-come-by samples such as patient biopsies, and is not limited by the 2-mm working distance of commonly used imaging systems.

**Figure 3:**
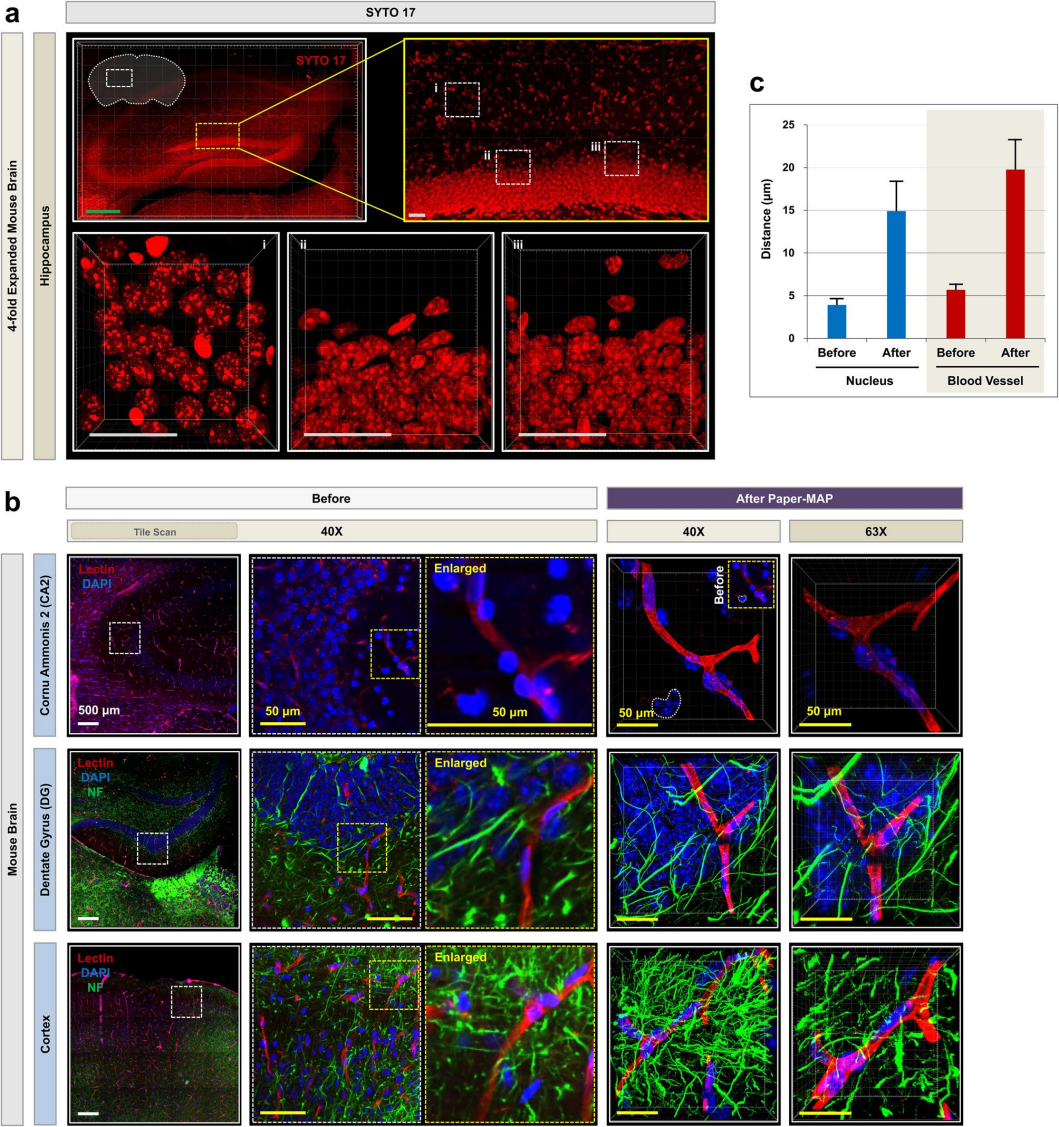
Visualization of vasculature and neural structure in rodent brains via Paper-MAP. (**a**) SYTO-17 immunostaining in the hippocampus of mouse brain sections processed via Paper-MAP, imaged with confocal microscopy (4 × 5 panels, horizontal × vertical tiles). Each image (enlarged; i, ii, and iii) was taken with the same 63 × objective lens and z-stacked (range: 100-μm) for comparison. Scale bars (green, 1000-μm; gray, 100-μm). (**b**) Lectin and neurofilament (NF) immunostaining in the hippocampus and cortex of mouse brain sections processed via Paper-MAP. Each image of before (left; unexpanded mouse brain sections of 4% PFA fixed) was taken with the same 40 × objective lens and single z-images (5 × 5 panels, horizontal × vertical tiles) for comparison. Enlarged images are of each regions (yellow box). Images of after Paper-MAP (right) was taken with the same 40 × and 63 × objective lens and z-stacked (range: 100-μm) for comparison. 3D projection of the blood vessel (lectin, red) and neurofilament (green) focusing on the cortex and hippocampus regions, including the cornu ammonis 2 (CA2), dentate gyrus (DG). Scale bars (white, 500-μm; yellow, 50-μm). (**c**) Quantification of size in nucleus (blue) and blood vessel (red) of the before (unexpanded) and after Paper-MAP in mouse brain. Quantification of total diameter from different ratio combinations. Data are presented as mean ± SD (standard deviation); n = 5 for each experimental group. Illustration of mechanism is visualized by PowerPoint v2016.

We performed immunostaining for SYTO 17 to visualize nuclear patterns in the mouse hippocampal sections processed via Paper-MAP (Fig. 3a). We then compared the morphological structures in the unexpanded 100 μm thick mouse brain sections of 4% PFA fixed (Before), and in those four-fold expanded mouse brain sections via Paper-MAP. To investigate the vasculatures and neural network in the mouse brains four-fold expanded via Paper-MAP, the mouse brain slices was pre-stained using DAPI, lectin dye and neurofilament (NF) antibody and was subsequently examined using confocal microscopy. As shown in Fig. 3b, we observed the mouse brain slice after clearing and expansion to visualize blood vessels and neuronal networks in the cortex region, and in cornu ammonis 2 (CA2) and dentate gyrus (DG) of the hippocampus region. Super-resolution Image analysis performed at 63 × and 40 × magnification revealed that the image sharpness of the nucleus, blood vessels and neurofilament was improved and that the images were enlarged more than four-fold. We were also able to quantify size of nucleus and blood vessel, which showed significant differences between before and fourfold expanded brain slices via Paper-MAP (Fig. 3c). This increase in magnification allowed for clear observation of cellular structures and neuronal networks in mouse brain using conventional confocal microscopy. These results demonstrate that our Paper-MAP technology is easy-to-use and rapidly imaging tool for the super-resolution imaging analysis of structural features in tissue slices.

### Visualization of hippocampal vasculature in Alzheimer’s disease via Paper-MAP

**Figure 4:**
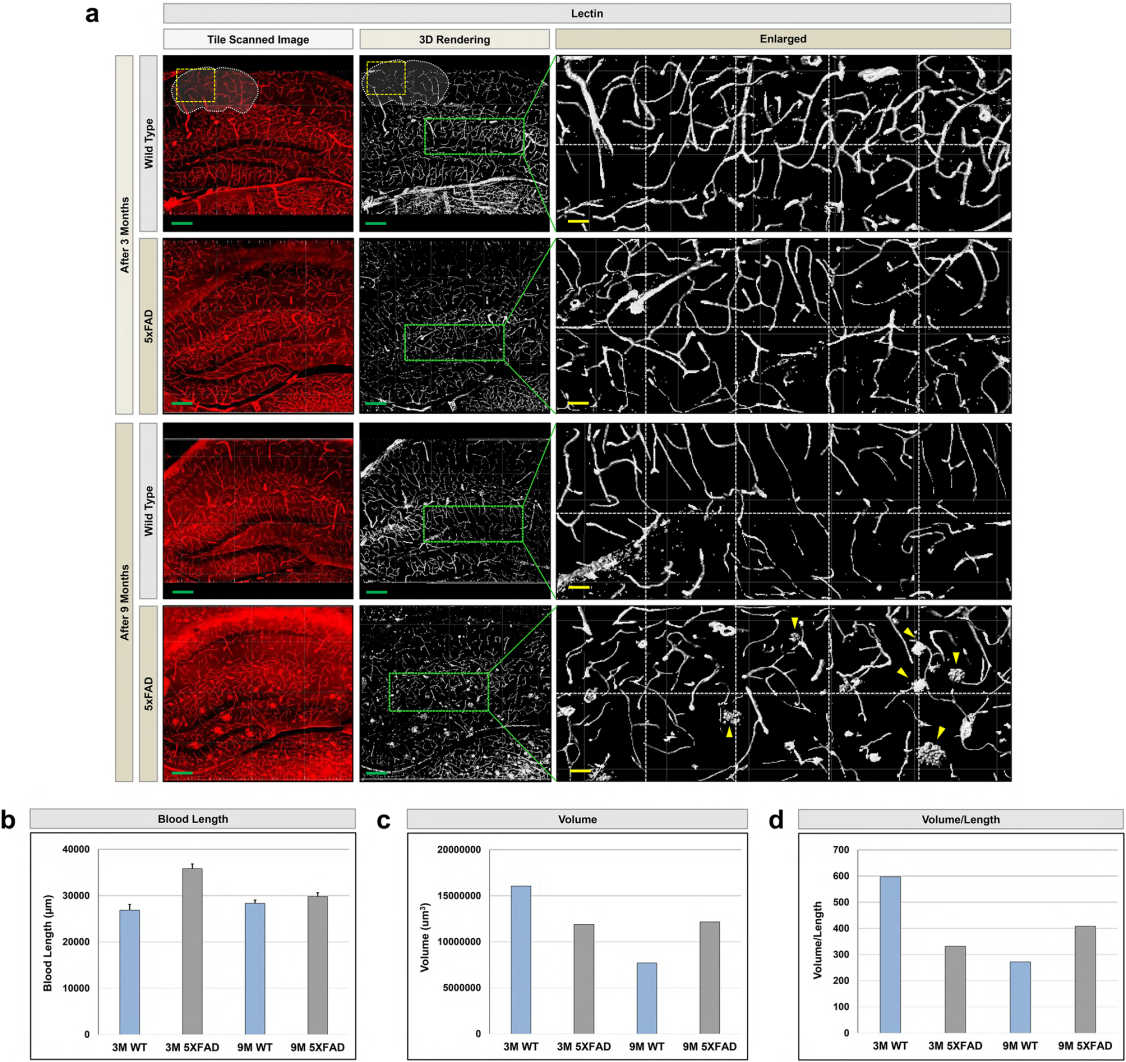
Visualization of hippocampal vasculature in rodent brains via Paper-MAP. (**a**) Lectin immunostaining of 5xFAD mouse brain tissues at 3 and 9 months compared to wildtype, processed via Paper-MAP. Enlarged photos (5 × 2 panels, horizontal × vertical tiles) are of hippocampal region. Yellow arrowheads indicate hemolytic plaques. Scale bars (green, 1000-μm; yellow, 100-μm). Quantification of blood vessel length (**b**), volume (**c**) and volume/length (**d**) in 5xFAD and wildtype hippocampus at 3 and 9 months. Quantification of total vessel length from different ratio combinations. Data are presented as mean ± SD (standard deviation); n = 5 for each experimental group. Illustration of mechanism is visualized by PowerPoint v2016.

To demonstrate proof-of-concept, we processed 100 µm-thick brain slices derived from the 5xFAD mouse model of Alzheimer’s disease (AD) according to our Paper-MAP protocol. AD brain slices were easily cleared and expanded by Paper-MAP within 1 day, after which we performed lectin immunostaining to investigate hippocampal angiogenesis. Vascular defects such as profuse thinning and increased microvasculature were apparent at 3 months of age, and by 9 months of age the AD hippocampus showed multiple hemolytic plaques (Fig. 4a). We were also able to quantify blood vessel length and volume, which showed significant differences between 5xFAD and wild-type brains (Fig. 4b–d). These results support the feasibility and efficacy of Paper-MAP as a tool for three-dimensional visualization of cellular and subcellular structures in intact tissue.

### Investigation of mouse spinal cord injury scar tissue via Paper-MAP

**Figure 5:**
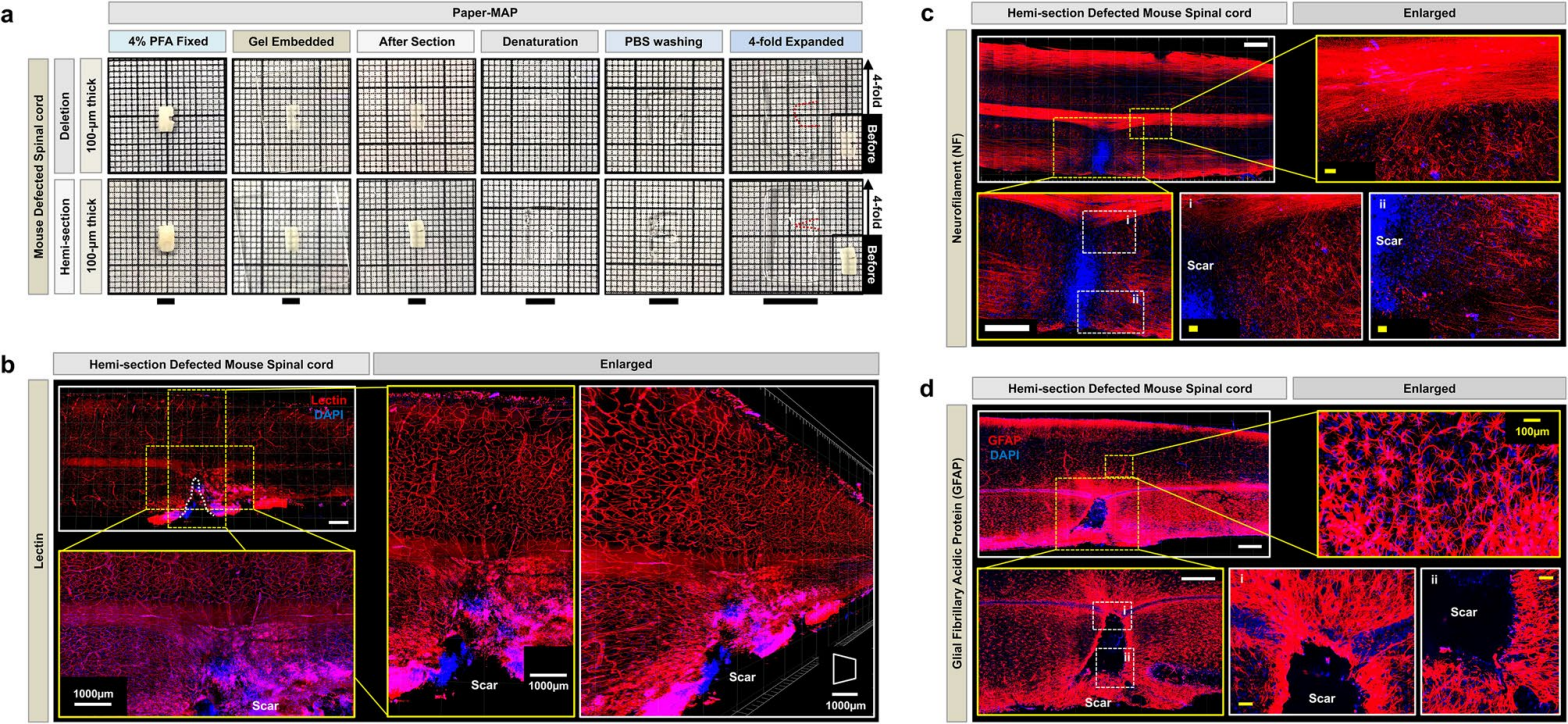
Paper-MAP processing of mouse spinal cord injury tissue. (**a**) Optical transparency of 100 μm-thick sections from mouse models of hemisection and deletion spinal cord injury models upon processing via Paper-MAP. Tissue transparency was assessed against a patterned background (length:width = 2 mm:2 mm). Representative images of samples from the hemisection spinal cord injury model processed via Paper-MAP and immunostained for lectin (red, range: 300-μm) (**b**), neurofilament (red) (**c**), and GFAP (red) (**d**). Yellow and white boxes indicate enlarged and 3D-projection images (range: 130-μm). Dotted white lines indicate scar region. Scale bars: white = 1000-μm, yellow = 100-μm. Illustration of mechanism is visualized by PowerPoint.

**Figure 6:**
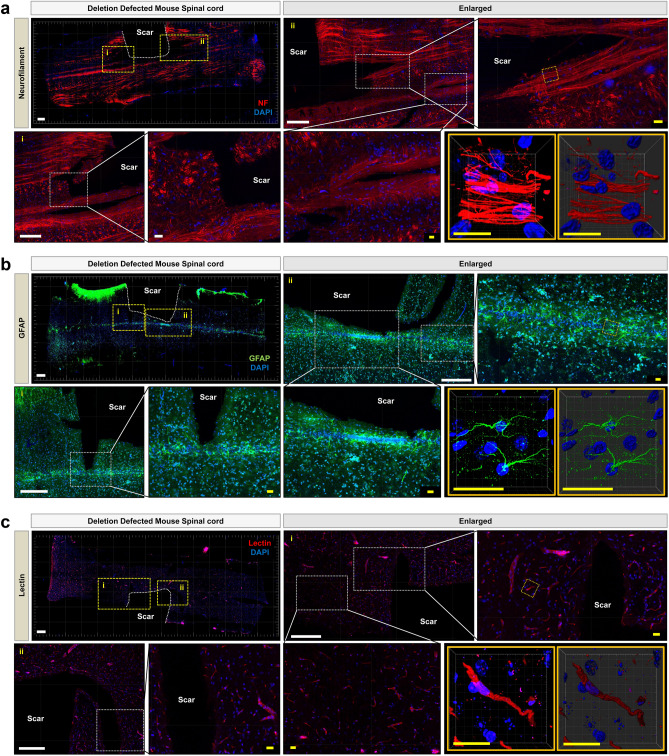
Paper-MAP processing of deletion spinal cord injury tissue in mouse. Representative tile-scanned images of samples from the deletion model of mouse spinal cord injury processed via Paper-MAP and immunostained for neurofilament (red) (**a**), GFAP (green) (**b**), and lectin (red) (**c**). Yellow and white boxes indicate enlarged and 3D-projection images (range: 100–150 μm). Dotted white lines indicate scar region. Scale bars: white = 1000-μm, yellow = 100-μm. Illustration of mechanism is visualized by PowerPoint v2016.

To further demonstrate the applicability of Paper-MAP, we applied this technique to two mouse models of spinal cord injury (SCI). Briefly, we generated two SCI models, involving spinal cord hemisection and deletion, respectively, and screened mice for poor performance on behavioral tests suggestive of injury (see Supplementary Figs. S1 and S2 online). We then harvested spinal cord tissue from injured mice and generated 100 µm-thick sections for processing via Paper-MAP (Fig. 5a). Immunostaining of hemisection-induced SCI for lectin revealed increased cell cohesion in the scar region, which was also observed upon immunostaining for neurofilament (NF-H, red) and astrocytic glial fibrillary acidic protein (GFAP, red) (Fig. 5b–d, Supplementary Video S2 online). Meanwhile, staining for neurofilament, GFAP, and lectin in deletion-induced SCI did not reveal increased cell cohesion in the scar region (Fig. 6a–c, Supplementary Videos S3–S6 online).

### Application of Paper-MAP to the histopathologic assessment of various rodent and human tissues

**Figure 7:**
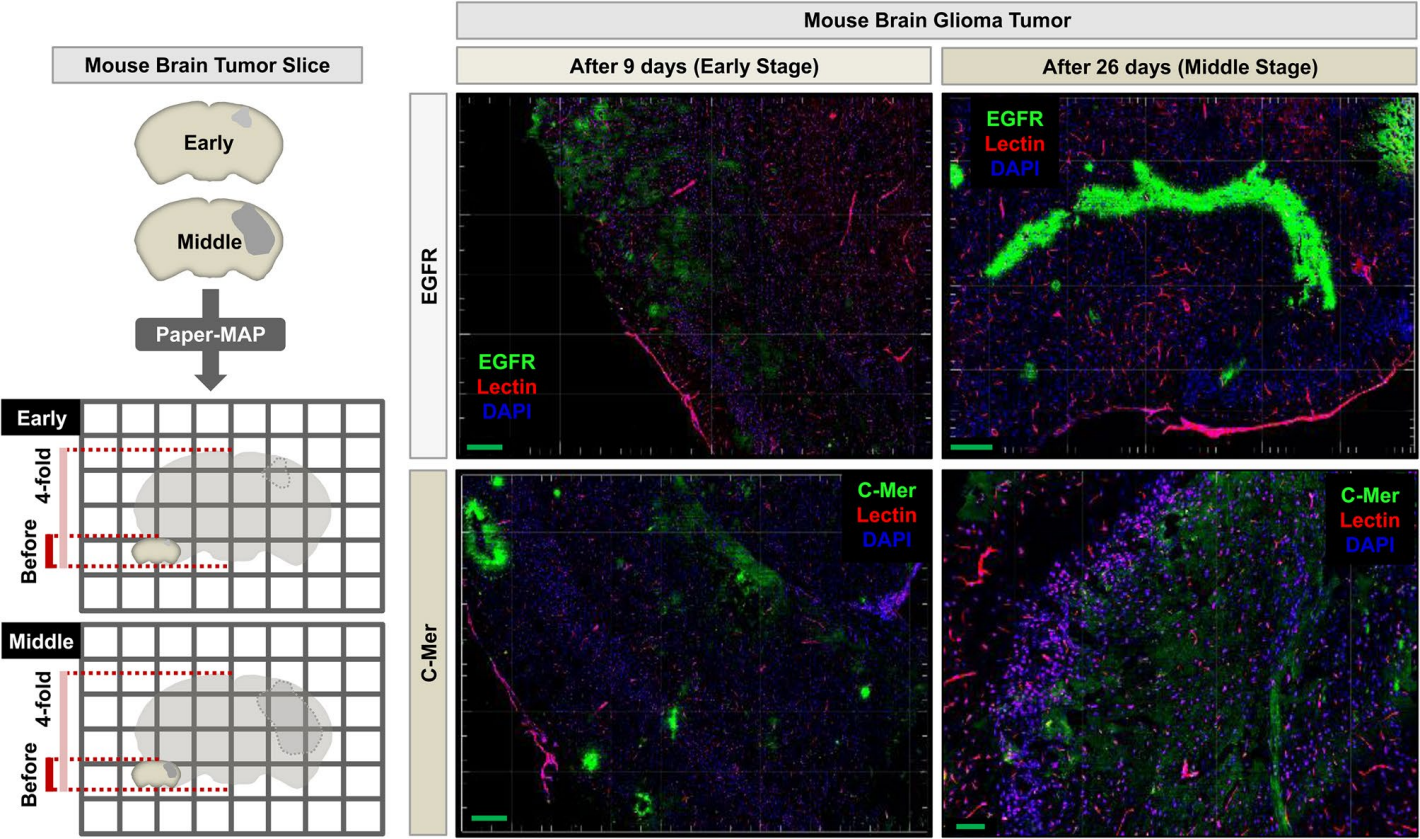
Application of Paper-MAP for tissue clearing and expansion in mouse brain tumor. Schematic representation of Paper-MAP processing using 100 μm-thick mouse brain tumor sections. Paper-MAP processing of human glioma xenografts upon 9 and 26 days post-transplantation in mice, followed by immunostaining for EGFR (green), c-Mer (green) and lectin (red). Scale bars: green = 2000-μm. Illustration of mechanism is visualized by PowerPoint v2016.

**Figure 8:**
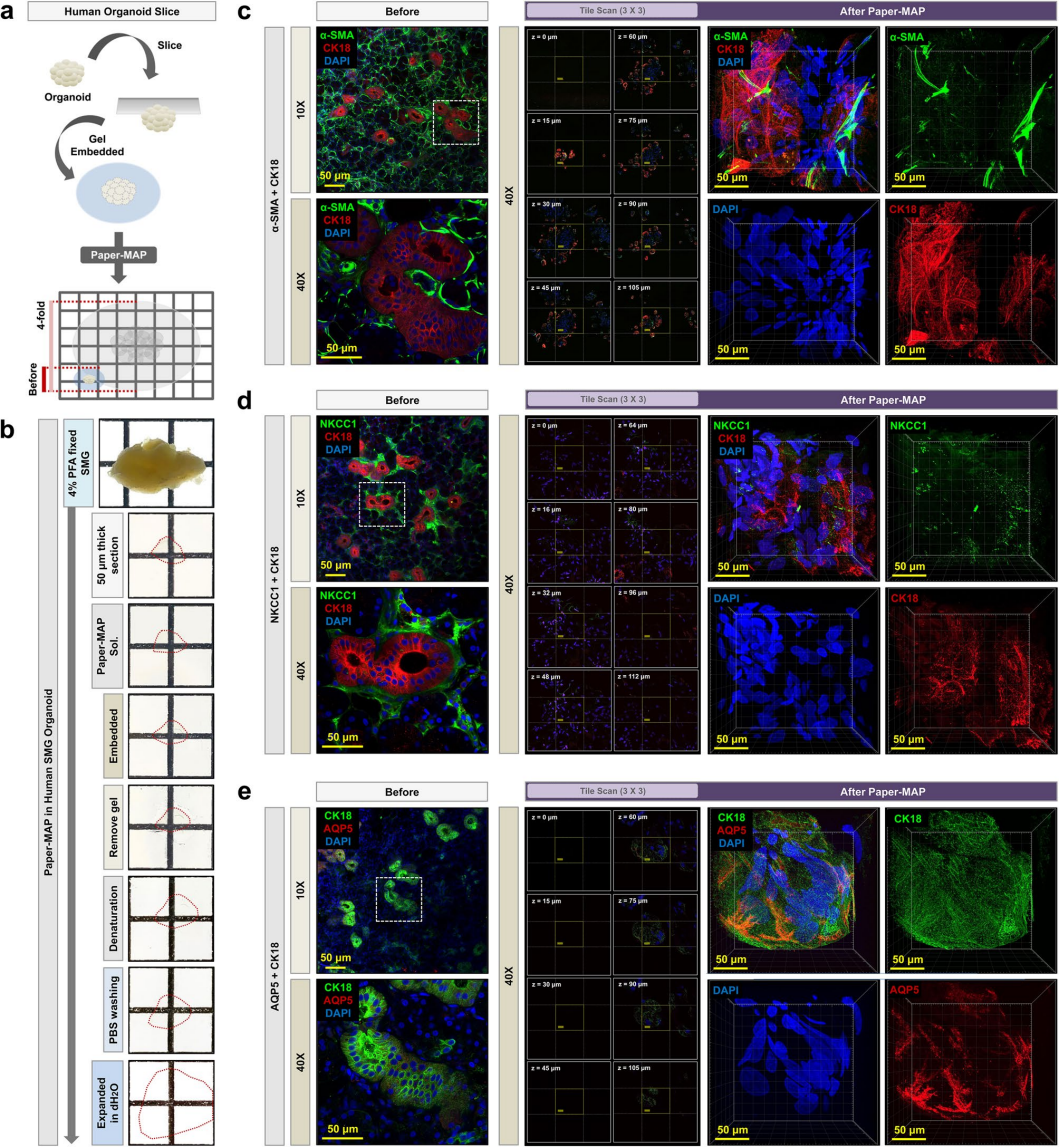
Application of Paper-MAP for tissue clearing and expansion in human submandibular gland organoid. (**a**) Schematic representation of Paper-MAP processing using 50 μm-thick human submandibular gland (SMG) organoid sections. (**b**) Optical transparency of human SMG organoid achieved via Paper-MAP. (**c-e**) Paper-MAP processing of human SMG organoids and subsequent immunostaining (range: 100-μm) for alpha-SMA (green), CK18 (red or green), NKCC1 (green), AQP5 (red) and DAPI (blue). Images of before (left, fixed in 4% PFA) and after Paper-MAP (right) was taken with the same 10 × and 40 × objective lens and z-stacked (range: 100-μm) for comparison. Scale bars: yellow = 50-μm. Illustration of mechanism is visualized by PowerPoint v2016.

We also sought to apply the Paper-MAP method to xenograft and organoid models to demonstrate its feasibility. We generated 100 µm-thick slices of U87MG brain glioma xenografts isolated at either the early (9 days post-transplantation) or middle stages (26 days post-transplantation) of tumor progression (Fig. 7), and performed immunostaining for epidermal growth factor (EGFR), proto-oncogene c-Mer Proto Oncogene Tyrosine Kinase (MERTK), and lectin. We observed a substantial increase in EGFR and c-Mer U87MG xenograft samples processed via Paper-MAP. We also performed Paper-MAP on slices generated from human submandibular gland (SMG) organoids, with subsequent immunostaining for alpha smooth muscle actin (α-SMA), Na–K-Cl cotransporter-1 (NKCC1), cytokeratin 18 (CK18) and aquaporin 5 (AQP5) (Fig. 8). Both tissues were easily cleared and remained intact for successful visualization with confocal microscopy.

**Figure 9:**
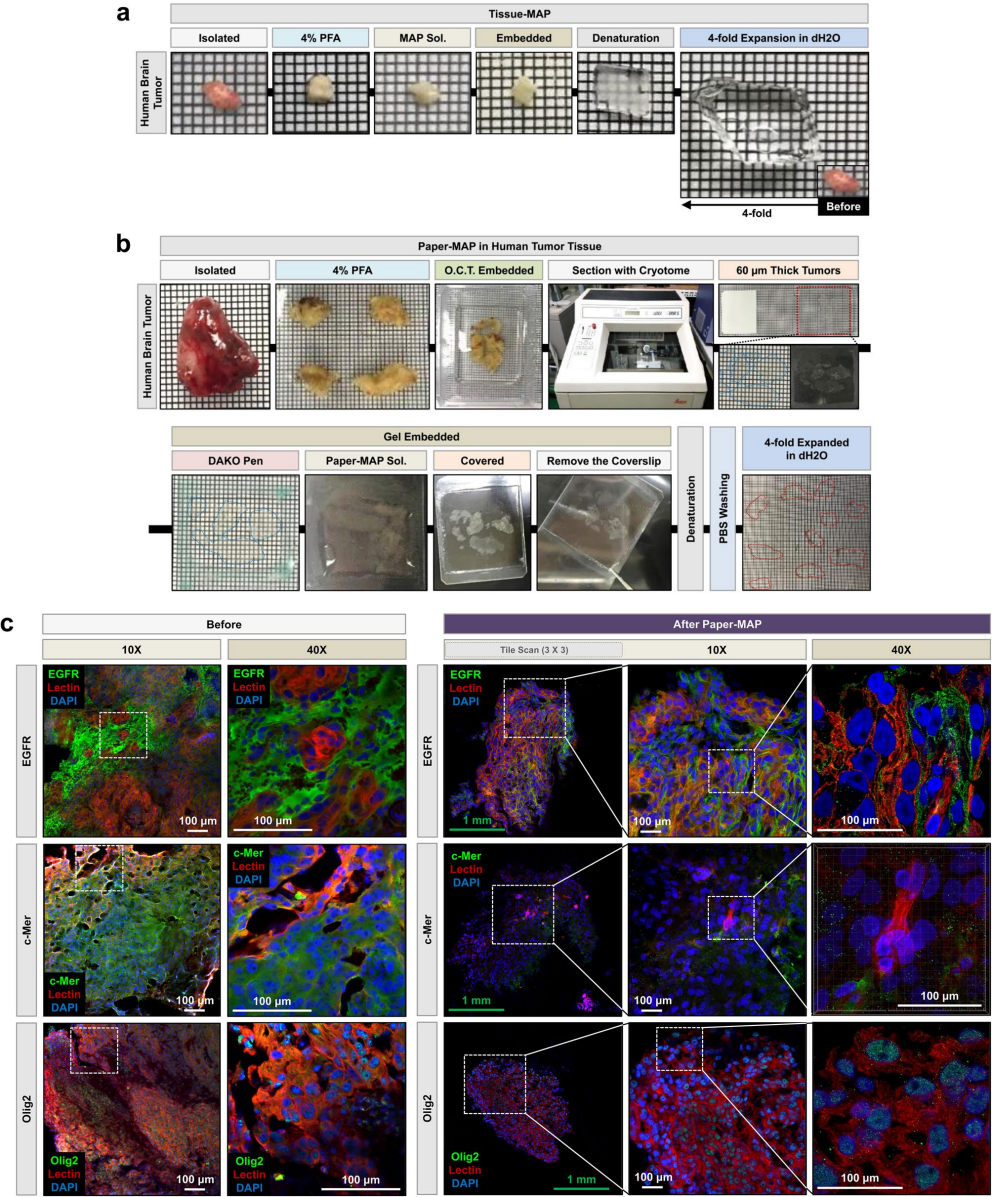
Application of Paper-MAP for tissue clearing and expansion in human brain tumor biopsy. (**a**) Optical transparency of human brain tumor biopsy achieved via Tissue-MAP. (**b**) Optical transparency of human brain tumor biopsy achieved via Paper-MAP. (**c**) Immunostaining for EGFR (green), Lectin (red), c-Mer (green), and Olig2 (green) in human brain tumor biopsy sections processed via Paper-MAP. Images of before (left, fixed in 4% PFA) and after Paper-MAP (right) was taken with the same 10 × and 40 × objective lens and z-stacked (range: 100-μm) for comparison. Scale bars: green = 1-mm, white = 100-μm.

Finally, to address the limitations of the original MAP protocol with regards to human biopsies, we performed Paper-MAP on a neurosurgical tumor biopsy, which was successfully cleared and expanded within 1 day. For comparison, we also performed the original MAP protocol, which cleared and expanded tumor tissue over a period of 10 days (Fig. 9a). As shown in Fig. 9b,c, there were no observable differences in the degree of tissue clearance achieved by either protocol, despite the significantly reduced incubation time of Paper-MAP. Immunostaining for epidermal growth factor receptor (EGFR), c-Mer proto oncogene tyrosine kinase, oligodendrocyte transcription factor 2 (Olig2), and lectin (blood vessel) of human brain tumor samples processed via optimized Paper-MAP also demonstrated the successful application of this technique to patient-derived samples.

## Discussion

In this study, we introduce Paper-MAP, a modified MAP technique that allows for the rapid clearing and expansion of tissues for three-dimensional ultrastructural imaging, within 2–3 days. During the time of its inception, the original MAP technique significantly advanced the ability to appreciate three-dimensional tissue architecture^9^. Its major limitations, however, included the long duration of time required for tissue processing, as well as the harsh effects of the denaturation and dissociation steps on tissue integrity, which require incubation at high temperatures without pre-treatment with crosslinking reagents to ensure tissue stability.

A major innovation of the Paper-MAP techniques is that they allow for the processing of 100 µm-thick sections as opposed to whole tissue, which enables the conservative use of rare samples that are difficult to obtain, such as patient biopsies. It is highly scalable, allowing for a single sample to be probed for multiple targets during subsequent immunostaining. The use of 100-µm sections as opposed to whole tissues also overcomes the limitation imposed by the 2-mm working distance of most imaging systems, rendering the need for a special, costly super-resolution microscope unnecessary. Other method for adjustment of tissue expending exists. Expansion microscopy (ExM) is a method to expand biological specimens (100-µm to 1-mm thick slices) with protease digestion of a hydrogel-tissue hybrid homogenizes the tissue’s mechanical characteristics, and allows four-fold linear expansion^10^. However the protease digestion step in ExM that can causes a loss of proteins, which limits the number of protein structures that can be imaged in the same sample.

Paper-MAP also uses an ammonium persulfate (APS) and tetramethyl ethylenediamine (TEMED)-based solution for embedding as opposed to the V50 cationic azo initiator used in the original MAP protocol, which reduces the time required for tissue embedding from over 3 days to 5 minutes^7,13^. Furthermore, whereas the original MAP protocol uses high denaturation temperatures as well as high concentrations of acrylamide (20–30%) to prevent tissue crosslinking, Paper-MAP involves the direct processing of tissue samples upon fixation in 4% PFA, which improves tissue stability. Fluorescence stability in sample is remains stable at room temperature and 37 °C in all incubation process containing clearing (denaturation) in previous study^13^. Whereas Paper-MAP (or original MAP) requires protein denaturation process of tissue at 95 °C, and it can be that some loss of labeled fluorescence signals. But this problem is possible surmountable problem to additional immunostaining at after Paper-MAP step, and it has no effect on the imaging analysis. To demonstrate the feasibility of Tissue-MAP (original MAP), we processed tissues harvested from mouse models of spinal cord injury according to our Tissue-MAP protocol, and performed immunostaining of lectin and DiD-D to visualize vasculature and neuronal networks, respectively. With the Tissue-MAP technique, we were able to successfully clear and expand injured spinal cord tissue such that we could observe blood vessel thinning and reduced neuronal density adjacent to the scar tissue, supporting its use for three-dimensional ultrastructural visualization of intact tissues. Nevertheless, Tissue-MAP required long processing times for tissue clearance, expansion, and subsequent immunostaining for super-resolution imaging, over a period of 20 days.

We then further optimized our Paper-MAP, which requires even shorter incubation times by removing the incubation step in low acrylamide (A.A) solution for 24 h. Paper-MAP allows for tissue clearance, expansion, and subsequent immunostaining in 2 days, compared to over 20 days for the original Tissue-MAP protocol. Using Paper-MAP, we observed vasculatures and neural structures with immunostaining of lectin dye and neurofilament antibodies in the cortex region, and in cornu ammonis 2 (CA2) and dentate gyrus (DG) of the hippocampus region using confocal microscopy. We also investigated hippocampal vasculature in the 5xFAD mouse model of Alzheimer’s disease (AD), compared to that of wildtype littermates. We observed age-dependent progression of disease, from blood vessel thinning and increased microvasculature apparent at 3 months to large, hemolytic plaques at 9 months^14^. Previous studies have suggested a role for blood-derived amyloid-β protein^15^, and while further research is required, our findings point to their potential role in these hemolytic plaques. Nonetheless, these results demonstrate the use of Paper-MAP to visualize disease pathology in a three-dimensional manner. Upon confirming the efficacy of Paper-MAP within the context of Alzheimer’s disease, we sought to apply the technique to a variety of pathological conditions, including two mouse models of spinal cord injury and a xenograft model of human glioma in mice. For spinal cord tissues, we specifically investigated angiogenesis, which is known to be defective in the early stages of spinal cord injury (SCI)^16,17^, as well as neuronal damage by immunostaining for lectin and neurofilament, respectively^18^. Consistent with previously established findings, we observed relatively high cell cohesion as well as tapering or disconnected neurofilaments and blood vessels near the scar region of hemisection-induced SCI tissues^19,20^. We also performed immunostaining of neurofilament, GFAP, blood vessels, γ-aminobutyric acid (GABA), parvalbumin, and tyrosine hydroxylase in deletion-induced mouse SCI tissues, which did not demonstrate such cell cohesion in scar tissue. In glioma xenograft-derived tissues, we observed the expression patterns of EGFR and c-Mer, which are known markers for cell proliferation and differentiation in tumors^21,22^.

Finally, we demonstrated the applicability of Paper-MAP to human tissues, including human submandibular gland (SMG) organoids, and more importantly, brain tumor biopsies. Another limitation of the original MAP protocol is that, because a key step involves the whole perfusion of the model organism with Tissue-MAP solution prior to harvesting the organ of interest, it is difficult to apply the technique to human tissues. Paper-MAP does not require whole perfusion and successfully clears tissues fixed in 4% PFA, which makes it amenable to patient derived biopsies. In sections of SMG organoids, we observed the expression patterns of alpha smooth muscle actin (α-SMA), Na–K-Cl cotransporter-1 (NKCC1), cytokeratin 18 (CK18) and aquaporin 5 (AQP5)^23–26^, and in the sections of human brain tumor biopsies, we assayed for the tumor-specific markers epidermal growth factor receptor (EGFR), c-Mer proto oncogene tyrosine kinase, and oligodendrocyte transcription factor 2 (Olig2)^27,28^.

In conclusion, Paper-MAP represent a significant advancement in the field of tissue clearing, as they allow for the clearing and subsequent visualization of individual tissue sections, in an unprecedented short amount of time. Given the demonstrated efficiency, efficacy, and versatility of the technique, Paper-MAP has strong potential for further elucidating the mechanisms underlying complex biological processes, especially with regards to the spatial relationships between cellular and subcellular structures that contribute to these phenomena.

## Materials and methods

### Human brain tumor samples

All study procedures were conducted in accordance with the Declaration of Helsinki. This study was approved by the Institutional Review Board (IRB) of Gangnam Severance Hospital, Yonsei University College of Medicine (IRB number: 3-2017-0232 Date of approval: 20 October 2017). The requirement for informed consent was waived by the IRB of Gangnam Severance Hospital because of the retrospective nature of this study. Fresh human brain tumor tissue samples were biopsied from neurosurgical patients operated on at the Gangnam Severance Hospital’s Department of Neurosurgery.

### Animal experiments

This study was carried out in strict accordance with the recommendations in the Guide for the Care and Use of Laboratory Animals of the Ministry of Agriculture, Food and Rural Affairs (MAFRA) and approved by the Institutional Animal Care and Use Committee (IACUC) of the Yonsei University College of Medicine (#2017–0230, Date of approval: 10 March 2020). All animal procedures were conducted under veterinarian supervision according to the guidelines imposed by the Ethical Committee. Mice used in these studies were cared for in accordance to the National Institutes of Health “Guide for the Care and Use of Laboratory Animals” and ARRIVE guidelines.

1. 5xFAD mouse model

2 week-old male 5xFAD (C57BL6) mice were at purchased from Central Lab. Animal Inc. (Seoul, Korea) and were raised in a specific pathogen free (SPF) environment.

2. Spinal Cord Injury (SCI) mouse model

8 week-old adult male ICR (Institute of Cancer Research) mice were purchased from Koatech Inc. (Gyeonggi-do, Korea). To induce spinal cord injury, mice were anesthetized with 2% isoflurane, and a dorsal laminectomy was performed at the C5 region of the spinal cord. The dura was removed using microscissors and forceps. In this study, we used two models of spinal cord injury. The lateral hemi-section model involved the use of a microblade in the C5 spinal cord region. In the deletion model, we used a microspatula to remove regions C4-C6 along the dorsal midline blood vessel. In both models, injuries were applied only to the right side of the C5 spinal cord region. Wounds were closed using a 4–0 black silk suture. All mice received 500 μL of sterile saline, cefazolin (25 mg/kg; Bristol Myers Squibb, New York, NY, USA), and buprenorphine (0.05 mg/kg; Reckitt and Colman Pharmaceuticals Inc., VA, USA) for 3 days after surgery. The workflow of for subsequent behavioral tests used to assess the two SCI models is in Supplementary Figs. S1 and S2 online. Mice exhibiting hand cannibalism and/or sudden death, and those that otherwise could not be evaluated with these behavioral tests, were excluded from the study.

3. Mouse orthotopic xenograft model for brain glioma tumor

6 to 8-week old male athymic nude mice were purchased from Central Lab. Animal Inc. (Seoul, Korea). 2 × 10^5^ dissociated U87MG cells (ATCC® HTB-14™; American Type Culture Collection, VA, USA) were implanted into the right frontal lobe of mice at a depth of 4.5 mm using a guide-screw system and Hamilton syringe. Gossypol (40 mg/kg; Sigma‐Aldrich Inc., St. Louis, MO, USA) and phenformin (100 mg/kg; Sigma‐Aldrich Inc., St. Louis, MO, USA) were orally administered daily. At either 9 or 26 days post-treatment, mice were euthanized and their brain tissues were harvested. If body weight decreased by more than 15% relative to baseline throughout the duration of the experiment, mice were euthanized according to the guidelines of the approved animal protocol. All experiments were performed as previously described^21^.

### Behavioral tests of SCI mouse models

Behavioral tests were first performed 2 days post-surgery, and tests were conducted weekly from 1 to 4 weeks after surgery. Forelimb recovery was assessed using the forelimb locomotor rating scale (FLRS)^29^, grip strength measurement (GSM)^30^, forelimb foot fault scoring (FFS)^31^, and the Irvine, Beatties, and Bresnahan (IBB)^32^ forelimb recovery scale. Hindlimb recovery was assessed using the Basso mouse locomotor scale (BMS), and hindlimb FFS^29,32,33^. In addition, body weight was measured 1 h before testing.

1. Forelimb locomotor rating scale (FLRS)

Mice were placed inside a transparent acrylic glass path, and mouse movements were recorded twice from the front and the back of the path by two experienced observers. Forelimb function recovery was assessed on a 17-point scale consisting of joint movement, weight support, stepping, predominant paw position, and toe clearance. The average time mice took to pass through the acrylic glass path was approximately 15 s.

2. Grip strength measurement (GSM)

Grip strength was measured using a grip strength meter (GSM, TSE Systems; SciPro Inc., London, United Kingdom). Mice were pre-trained three times a week for two weeks. Paper tape was placed on a single forepaw, and the maximum force of grip strength was measured by pulling the tail after the free paw without paper tape caught the GSM bar. Grip strength was tested three times per week for 6 weeks post-injury.

3. Foot fault scoring (FFS)

The FFS test was performed using a video-recorded ladder rung walking task. The rung walking apparatus was consisted of two Plexiglas walls (70 cm × 15 cm) with 0.12 cm diameter holes at 5 cm intervals. The holes were filled with 8-cm metal bars (diameter 0.1 cm). Foot fault was scored using the following scale: 0 point (total miss), 1 point (deep slip), 2 points (slight slip), 3 points (replacement), 4 points (correction), 5 points (partial placement) and 6 points (correct placement).

4. Irvine, Beatties, and Bresnahan (IBB) forelimb recovery scale

During the test, mice were given two minutes to eat circle-shaped cereal in a transparent acrylic glass cylinder (10-cm diameter) with glass mirrors on each side. Forelimb recovery was assessed via the following: predominant elbow position, proximal forelimb movement, contact non-volar support, predominant forepaw position, contact volar support, cereal adjustments, wrist movement, contact digit movements, and grasping method. Mice were also scored based on the original IBB scale, which consists of predominant elbow position, forepaw position, cereal adjustments, digit movements, and grasping method. Mice were acclimated to the testing environment 2 weeks prior to surgery, during which they were given a cereal diet within the cylinder used in the test.

5. Basso mouse locomotor scale (BMS)

Basso mouse locomotor scale (BMS) testing was performed simultaneously with FLRS under similar conditions. After taking two video recordings per animal, FLRS and BMS were verified by video evaluation. In BMS, the scores were evaluated based on 9-point scale consisting of ankle movement, plantar stepping, coordination, paws parallel, trunk stability, and tail movement.

### Injured lesion preparation and quantification

At each experimental endpoint, mice were anesthetized with an overdose of zoletil (Virbac, Carros, France) and rompun (Bayer HealthCare, Leverkusen, Germany). The thorax was exposed, and an incision was made in the right atrium of the heart. Trans-cardiac perfusion was performed with equal volumes of ice-cold 0.1 M PBS and 4% paraformaldehyde (PFA) using a 50 mL syringe. After fixation for 1 day in 4% PFA, the solution was replaced with 30% sucrose in 0.1 M PBS. A 3-mm sample of the spinal cord was then harvested without damage to the injury site, which was further processed into 20 µm-thick sections using a cryostat. To analyze the volume of the spinal cord lesion, sections were stained with hematoxylin (Sigma‐Aldrich Inc., St. Louis, MO, USA) and eosin (Sigma‐Aldrich Inc., St. Louis, MO, USA), and the volume was quantified using ImageJ software (National Institutes of Health, MD, USA).

### Cell culture

U87 spheres were generated from the U87MG cell line (ATCC® HTB-14™; American Type Culture Collection, VA, USA). Cells were cultured in media, composed of DMEM/F12 (Life Technologies Co., Carlsbad, CA, USA), 10% fetal bovine serum (FBS; Life Technologies Co., Carlsbad, CA, USA), 1 × B27 (Invitrogen Inc., Carlsbad, CA, USA), 20 ng/mL basic fibroblast growth factor, and 20 ng/mL epidermal growth factor (Sigma-Aldrich Inc., St. Louis, MO, USA).

### Human salivary gland organoid culture

Human single clonal stem cells were used to establish salivary gland organoids, as previously described^26^. Cells were seeded in a petri dish coated with 1% of Pluronic F127 (Sigma-Aldrich Inc., St. Louis, MO, USA) in Phosphate-buffered saline (PBS) at a density of 40,000 cells/cm^2^. Suspended cells were cultured in low glucose DMEM media (Life Technologies Co., Carlsbad, CA, USA) supplemented with 10% FBS and 100 U/ml streptomycin-penicillin (Invitrogen Inc., Carlsbad, CA, USA). After 7 days of culture, the established salivary gland organoid was post-treated to suit the purpose of the experiment.

### MAP technique

Tissue clearing, denaturation, and expansion were performed according to previously established protocols^9^, which were specifically optimized for the clearing and expansion of whole intact tissues.

1. Perfusion of experimental mouse

Mice were anesthetized with 2% isoflurane. Upon opening the thorax, an incision was made in the right atrium of the heart. Trans-cardiac perfusion was performed with equal volumes of ice-cold 0.1 M PBS and 4% PFA solution or either Tissue-MAP solution (30% acrylamide (Sigma‐Aldrich Inc., St. Louis, MO, USA), 0.1% bis-acrylamide (Bio-Rad Laboratories Inc, Hercules, CA, USA), 10% sodium acrylate (Sigma‐Aldrich Inc., St. Louis, MO, USA), 0.05% photoinitiator V-50 (Wako Chemicals, Richmond, VA, USA) in 0.1 M PBS), using a 50 mL syringe. The tissue was harvested following standard protocol^5,6,34^.

2. Tissue-MAP

Samples fixed in Tissue-MAP were incubated in low acrylamide (A.A) solution (4% acrylamide and 4% PFA in 0.1 M PBS) at 37 °C for 1 day under light protection. The tissue was then washed for 3 h with 0.1 M PBS, then incubated in inactivation solution (1% acetamide (Sigma‐Aldrich Inc., St. Louis, MO, USA), 1% glycine (Sigma‐Aldrich Inc., St. Louis, MO, USA), 0.02% sodium azide (Sigma‐Aldrich Inc., St. Louis, MO, USA) in dH_2_O, pH 9.0) at 37 °C for 3 days. After inactivation, the sample was immersed in Tissue-MAP solution at room temperature for 3 days in a shaking incubator. The sample was embedded with nitrogen gas using Easy-Gel (LifeCanvas Technologies, Boston, MA, USA) at 45 °C for 2–4 h, and then transferred to denaturation solution (200 mM sodium dodecyl sulfate [SDS; Affymetrix Inc., Santa Clara, CA, USA], 200 mM sodium chloride [NaCl; Sigma‐Aldrich Inc., St. Louis, MO, USA], 50 mM Tris [Affymetrix Inc., Santa Clara, CA, USA], in dH_2_O, pH 9.0). Subsequently, the tissue was incubated at 90 °C in denaturation solution for 2–3 days using EasyClear (LifeCanvas Technology, MA, USA). The denatured tissue was washed three times for 3 h in 0.1 M PBS. The sample was transferred to dH_2_O and incubated at room temperature until it was expanded four-fold. Further details are provided in Fig. 1a.

3. Paper-MAP

Samples fixed in 4% PFA were sliced into 100 μm thick sections using a Leica VT1000-S vibratome (Leica biosystems, Wetzlar, Germany). Sections were transferred to 0.1 M PBS and washed at room temperature for 30 min. Brain sections were washed three times in 0.1 M PBS for 3 h. Samples were transferred onto a 24 × 60 mm coverslip and embedded into a hybrid polymer by adding Paper-MAP solution (20% acrylamide, 10% sodium acrylate, 0.1% bis-acrylamide, 0.65% TEMED [Sigma‐Aldrich Inc., St. Louis, MO, USA], in 0.1 M PBS), which was left to polymerize for 5 min. Samples were then treated with freshly prepared 5% ammonium persulfate (APS; Sigma‐Aldrich Inc., St. Louis, MO, USA), incubated in denaturation solution at 90 °C for 9–12 h, and washed in 0.1 M PBS. Finally, samples were transferred to dH_2_O until they expanded fourfold in size. Further details are provided in Fig. 1b.

### Paper-MAP for human SMG organoids

Human submandibular gland (SMG) organoids were fixed with 4% PFA for 1 h and washed three times for 3 h with 0.1 M PBS. Organoids were embedded into a MAP hybrid polymer using Paper-MAP solution, followed by treatment with freshly prepared 5% APS. Gels were sliced into 100 µm sections and incubated in denaturation solution at 95 °C for 30 min. Sections were washed three times for 3 h in 0.1 M PBS, and incubated at room temperature in dH_2_O until they expanded fourfold in size.

### Paper-MAP for human brain tumor samples

4% PFA fixed human brain tumor samples were embedded in O.C.T. compound (Sakura Finetek USA Inc., CA, USA) at –20 °C. Samples were processed into 60 µm sections using a Leica CM1850 cryostat microtome (Leica biosystems, Wetzlar, Germany). Sections were treated with Paper-MAP solution and 5% APS, then incubated in denaturation solution at 90 °C for 9–12 h. Upon washing in 0.1 M PBS, sections were transferred to dH_2_O until they expanded fourfold in size. Further details are provided in Fig. 7d.

### Immunostaining and imaging

After tissue clearance and expansion, samples were blocked with 2% bovine serum albumin (BSA; Sigma‐Aldrich Inc., St. Louis, MO, USA) in 0.1 M PBS for 6 h and treated with primary antibodies for 1–4 days at room temperature. Samples were washed 3 times in PBST (0.1% Triton X‐100 [Sigma‐Aldrich Inc., St. Louis, MO, USA] in 0.1 M PBS) for 3–24 h and incubated with conjugated secondary antibodies for 1–4 days. All antibodies and dyes used in this study are listed in Supplementary Table S1 online.

Prior to imaging, tissues were re-expanded in dH_2_O at room temperature for 1–24 h. Intact whole tissue samples were placed on a slide inside a U-shaped Blu-Tack adhesive (Bostik, WI, USA), and covered with a glass-bottom Wilco or confocal dish filled with dH_2_O. Samples of Paper-MAP can long term storage in dH_2_O for until a month at room temperature. Fluorescence-labeled samples of Paper-MAP can long term storage for 1–2 months with shrinkage form in refractive index matching solutions (EasyIndex; LifeCanvas Technologies, Cambridge, MA, USA). All clear images were captured using iPhone-X camera (Apple Inc., Cupertino, CA, USA). Neural fiber and blood vessel images of whole spinal cord tissue were obtained with a LaVision Light-sheet Ultramicroscope (LaVision BioTec GmbH, Bielefeld, Germany) at 2.0 × (0.5 NA) magnification. All images of Paper-MAP were captured using a confocal microscope (LSM780 and LSM980; Carl Zeiss, Oberkochen, Germany) at a magnification of 10 × (0.45 NA, 2.0 mm working distance), 20 × (0.8 NA), and 40 × (0.8 NA). Images were processed and analyzed using Zeiss ZEN-2 software (Carl Zeiss, Oberkochen, Germany), and results were processed into three-dimensional images and videos using Imaris v8.0.1 software (Bitplane, Belfast, United Kingdom).

### Quantification of vascular architecture

We compared the lengths, density, and volume fraction of the hippocampal vessels between several sub-regions including the three cornu ammonis (CA1, CA2, CA3) regions and dentate gyrus (DG) in wild type and 5xFAD brains. Upon performing immunostaining for lectin, blood vessel length and volume were quantified using Imaris v8.0.1 software. Images were reconstructed with “Surpass view” on the “Surface” icon in the objects toolbar. To analyze the Region of Interest (ROI), 10 ROIs were selected from each sub-region. “Channel” was used as the source channel, and “Absolute Intensity” was used to adjust thresholds. Results were filtered by the number of voxels using the “Classify Surfaces” tab, according to default settings. Quantification was performed using the “Statistics” tool.

## Supplementary Information

Supplementary Information.

Supplementary Video S1.

Supplementary Video S2.

Supplementary Video S3.

Supplementary Video S4.

Supplementary Video S5.

Supplementary Video S6.
