# Supplementary material for: A novel paper MAP method for rapid high resolution histological analysis
Source: Sci Rep. 2021 Dec 2;11:23340. doi: 10.1038/s41598-021-02632-1 (PMC8639998; doi:10.1038/s41598-021-02632-1)
Supplement: Supplementary file 1 — Supplementary Information. [file 41598_2021_2632_MOESM1_ESM.pdf]

SUPPLEMETARY INFORMATION

A Novel Paper MAP Method for Rapid High Resolution Histological Analysis

Authors: Mirae Lee [1,2,3,†], Jiwon Woo [2,3,4,†], Doh-Hee Kim [5,†], Yu-Mi Yang [1,2,3], Eunice Yoojin Lee [8], Jung-Hee Kim [5], Seok-Gu Kang [6,7], Jin-Kyung Shim [6], and Jeong Yoon Park [1,2,3,*]

Affiliations:

[1] Department of Neurosurgery, Graduate School of Medical Science, Brain Korea 21 Project, Yonsei University College of Medicine, Seoul, 03722 Republic of Korea

[2] The Spine and Spinal Cord Institute, Department of Neurosurgery, Gangnam Severance Hospital, Yonsei University College of Medicine, Seoul, 06273 Republic of Korea

[3] Biomedical Research Center, Gangnam Severance Hospital, Yonsei University College of Medicine, Seoul, 06273 Republic of Korea

[4] Biomedical Research Institute, Biohedron Therapeutics Co., Ltd., Seoul, 06273 Republic of Korea

[5] Research Institute, Seoul Medical Center, Seoul, 02053 Republic of Korea

[6] Department of Neurosurgery, Brain Tumor Center, Severance Hospital, Yonsei University College of Medicine, Seoul, 03722 Republic of Korea

[7] Department of Medical Sciences, Yonsei University Graduate School, Seoul, 03722 Republic of Korea

[8] Columbia University Vagelos College of Physicians and Surgeons, New York, NY, 10032 USA

[†] Mirae Lee, Jiwon Woo, and Doh-Hee Kim. These authors contributed equally to this work.

* Corresponding author:

Prof. Jeong-Yoon Park (M.D., Ph.D.), E-mail: spinepjy@yuhs.ac

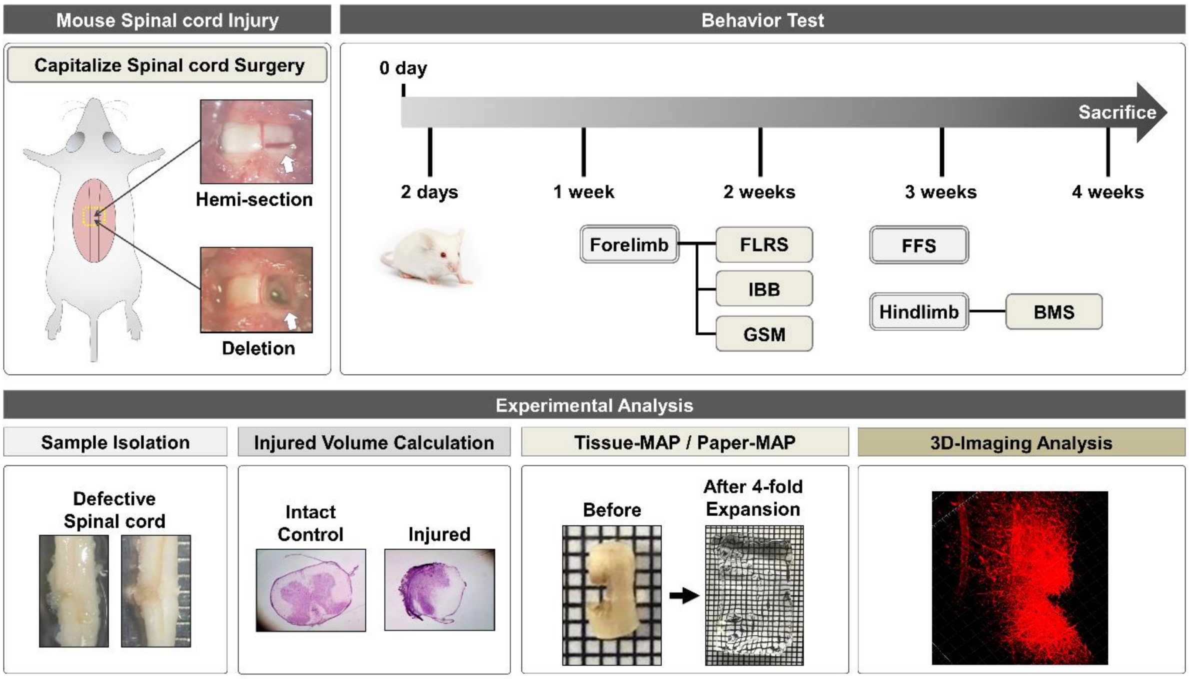

Supplementary Figure S1. Schematic of spinal cord injury models, subsequent behavioral assessment and Tissue-MAP/Paper-MAP processing.

This study used two methods of mouse spinal cord injury, the hemisection model and the deletion model. Mice of ecah model were evaluated by a series of behavioral tests for four weeks post-injury. Mice that displayed signs of spinal cord injury were euthanized and their spinal cord was isolated at the site of injury for processing via either Tissue-MAP or Paper-MAP, followed by immunostaining and image analysis.

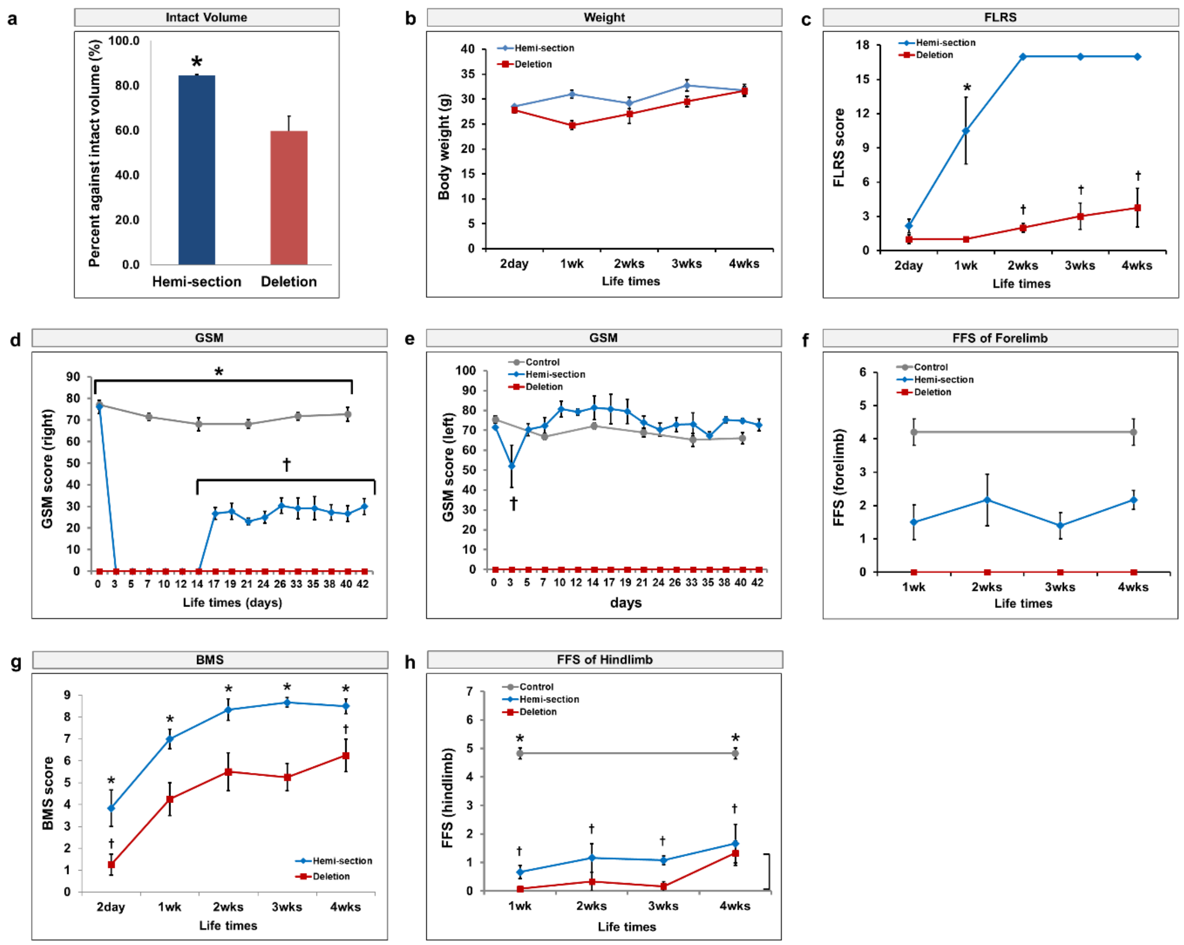

Supplementary Figure S2. Behavioral tests in mouse spinal cord injury models. (a) Quantification of injury volumes of H&E stained spinal cord sections from hemisection and deletion SCI models. Injury volume was described as a percentage of the volume of the intact spinal cord. **(b)** Quantification of body weight in hemisection and deletion SCI models for 4 weeks post-injury. **(c)** Evaluation of forelimb recovery via FLRS. **(d)** Evaluation of left grip strength via GSM. **(e)** Evaluation of right grip strength via GSM. The value of grip strength is the maximum force recorted among 10 repeated trials. **(f)** Evaluation of forelimb recovery via FFS. **(g)** Evaluation of hindlimb recovery via BMS. **(h)** Evaluation of hindlimb recovery via FFS. All values are presented as the mean ± SD.* indicates $p < 0.05$.

Supplementary Table S1. Antibodies and dyes used in this study.

Type	*Name*	*Cat. No.*	*Company*
Primary antibody	Anti-Neurofilament	837904	biolegend
	Anti-GFAP	835301	biolegend
	Anti-GABA B receptor 1	ab55051	abcam
	Anti-Parvalbumin	ab94362	abcam
	Anti-Tyrosine hydroxylase	ab112	abcam
	Epidermal Growth Factor Receptor (EGFR)	PAA757Hu02	Cloud-Clone Corp.
	C-mer Proto Oncogene Tyrosine Kinase (MERTK)	PAE271Hu01	Cloud-Clone Corp.
	Anti-Cytokeratin 18	ab668	abcam
	Anti-Aquaporin 5	AQP-005	almone labs
	Anti-Olig2	H0010215-M03	Abnova
	Anti-alpha Smooth Muscle Actin (SMA)	ab5694	abcam
	Anti-NKCC1	ab59791	abcam
Secondary antibody	Donkey Anti-Mouse IgG H&L (Alexa Fluor® 488)	ab150109	abcam
	Goat Anti-Mouse IgG H&L (Alexa Fluor® 647)	ab150115	abcam
	Goat Anti-Rabbit IgG H&L (Alexa Fluor® 488)	ab150077	abcam
	Donkey Anti-Rabbit IgG H&L (Alexa Fluor® 647)	ab150063	abcam
Dye	Lycopersicon Esculentum (Tomato) Lectin (LEL, TL), DyLight® 594	DL-1177	Vector Laboratories
	DiD' oil; DiIC18(5) oil	D307	invitrogen
	SYTO™ 17 Red Fluorescent Nucleic Acid Stain	S7579	invitrogen

SUPPLEMENTARY VIDEO LEGENDS

Supplementary Video S1. 3D rendering of vasculature in deletion mouse SCI tissue processed via Tissue-MAP.

Sections derived from the deletion SCI mouse model were processed with Tissue-MAP and stained with tomato lectin dye. Images were obtained using 2.0× (0.5 NA) magnification on a LaVision Ultra Microscope. Results were reconstructured using Imaris software.

Supplementary Video S2. 3D rendering of vasculature in hemisection mouse SCI tissue processed via Paper-MAP.

Sections derived from the hemisection SCI mouse model were processed with Paper-MAP and stained with tomato lectin dye. Images were obtained using 10× (0.45 NA) magnification on a LSM780 confocal microscope. Results were reconstructured using Imaris software.

Supplementary Video S3. Neurofilament immunostaining in deletion mouse SCI tissue processed via Paper-MAP (10×).

Sections derived from the deletion SCI mouse model were processed with Paper-MAP and immunostained for neurofilament. Images were obtained using 10× (0.45 NA) magnification on a LSM780 confocal microscope. Results were reconstructured using Imaris software.

Supplementary Video S4. Neurofilament immunostaining in deletion mouse SCI tissue processed via Paper-MAP (40×).

Sections derived from the deletion SCI mouse model were processed with Paper-MAP and immunostained for neurofilament. Z-stack images were obtained using 40× (0.8 NA) magnification on a LSM780 confocal microscope. Results were reconstructured using Imaris software.

Supplementary Video S5. GFAP immunostaining in deletion mouse SCI tissue processed via Paper-MAP (10×).

Sections derived from the deletion SCI mouse model were processed with Paper-MAP and immunostained for GFAP. Z-stack images were obtained using 10× (0.45 NA) magnification on a LSM780 confocal microscope. Results were reconstructured using Imaris software.

Supplementary Video S6. GFAP immunostaining in deletion mouse SCI tissue processed via Paper-MAP (40×).

Sections derived from the deletion SCI mouse model were processed with Paper-MAP and immunostained for GFAP. Z-stack images were obtained using 40× (0.8 NA) magnification on a LSM780 confocal microscope. Results were reconstructured using Imaris software.
